# Giant Ectopic Parathyroid Adenoma Arising in the Posterior Mediastinum. Report of Case and a Review

**DOI:** 10.1155/2022/6473197

**Published:** 2022-11-09

**Authors:** Yousef S. Amr, Mousa M. Saleh, Samir S. Amr

**Affiliations:** ^1^Department of Surgery, Istishari Hospital, Amman, Jordan; ^2^Department of Pathology and Laboratory Medicine, Istishari Hospital, Amman, Jordan

## Abstract

We present a case of a greatly enlarged giant ectopic parathyroid adenoma that weighed 43 grams, which was located in the posterior mediastinum of a 74-year-old man. The patient presented with generalized weakness and decreased level of consciousness. He was found to have elevated level of serum calcium (19.9 mg/dl), and a subsequent assay of parathyroid hormone (PTH) was greatly elevated (2234 pg/ml). We report the course of management and outcome of the patient and present a review of the literature on giant ectopic parathyroid adenomas in the posterior mediastinum.

## 1. Introduction

Parathyroid adenomas (PA) usually arise in any of the four parathyroid glands located in their usual anatomic location in the neck, close to the thyroid gland. In some instances, they can arise in ectopic locations including intrathyroidal, intrathymic, mediastinal, submandibular, and within the carotid sheath [1]. Their usual weight usually does not exceed 3 grams. However, when they weigh over 3.5 grams, they are labeled as giant adenomas.

We present herein a patient who presented to the emergency department with generalized weakness and decreased level of consciousness. Laboratory workup revealed hypercalcemia. This prompted the measurement of PTH which was significantly elevated. ^99m^Tc-sestamibi scintigraphy and chest computerized tomography (CT) scan revealed a markedly enlarged posterior mediastinal parathyroid adenoma that was excised through open thoracotomy. The tumor was paratracheal on CT scan, a location that was reported only twice for such tumor. When excised, the tumor was found between vena cava, azygos vein and the spine, a location not reported previously. We discuss the surgical approach for such tumors, and the difficulties in excising them and causes of repeated surgical interventions. We reviewed previously reported cases of ectopic posterior mediastinal giant PAs.

## 2. Case Report

A 74-year-old male presented with generalized weakness, decreased level of consciousness, poor oral intake, ataxia, dizziness, and disorientation in April 2020. Complete blood count revealed elevated white blood cells at 15.1 × 10^3^/cubic mm. ESR was elevated at 30 mm/H (normal range: 0.00–15.00). Sodium, potassium, and chloride levels were normal. Random blood sugar was 136 mg/dl (normal range: 70-150). Urea and creatinine serum levels were both elevated at 72 mg/dl (normal range: 15-45) and 1.41 (normal range: 0.70-1.20), respectively. He had elevated calcium level at 19.9 mg/dl (normal range: 8.90-10.70). Phosphorus level revealed mild elevation at 5.2 mg/dl (normal range: 2.50–5.00). Liver function tests were within normal limits. Parathyroid hormone (PTH) level was markedly elevated at 2234 pg/ml (normal range: 15.00–65.00). Due to the marked elevations of calcium and PTH level, he was admitted to the hospital for further investigation and management of hyperparathyroidism.

Radiological investigations revealed unremarkable thyroid and pelvic ultrasound studies. Brain computed tomography (CT) scan displayed no intracranial hemorrhage, a few bilateral periventricular white hypodensities and ischemic small vessel disease. Chest CT scan exhibited a 6 × 4 × 3 cm well-defined lobulated heterogeneous mediastinal right paratracheal mass suggestive of neoplastic process, including enlarged lymph node or lymphoma. The possibility of a giant mediastinal ectopic parathyroid adenoma could not be ruled out based on elevated serum levels of calcium and PTH. Both lung fields appeared grossly unremarkable.

Due to the marked elevations of calcium and PTH level, he was treated with zoledronic acid 4 mg intravenously over 30 minutes, Lasix 80 mg intravenously, along with intravenous fluids. On the following day, calcium level dropped to 15.1 mg/dl. On his discharge, three days later, his calcium level was at 9.8 mg/dl.

**Figure 1:**
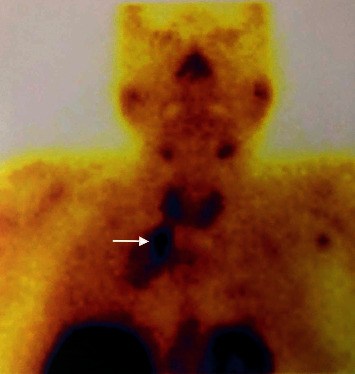
Radionuclide parathyroid scintigraphy with 99mTc-sestamibi scan demonstrates focal abnormal persistent uptake inferior to the right thyroid lobe and extending into the mediastinum (arrow).

Radionuclide parathyroid study with ^99m^Tc-sestamibi scintigraphy scan was done at another facility after discharge, and it demonstrated focal abnormal persistent uptake inferior to the right thyroid lobe and extending into the mediastinum, correlating to the site of the previously diagnosed right paratracheal mass lesion (Figure 1). It showed irregular tracer retention with areas of low uptake probably representing necrotic parts of the large mass. These findings were consistent with a right lower parathyroid lesion extending into the mediastinum. Considering the age of the patient, the irregularity in the uptake, the large size and the very high PTH levels, and the possibility of parathyroid carcinoma was raised.

Patient was then admitted to the surgical unit in May 2020 for the removal of the ectopic posterior mediastinal parathyroid tumor. Laboratory tests prior to the surgery showed calcium level to be 11.0 mg/dl and PTH at 1103 pg/ml. The patient was put under general anesthesia. A single port thoracoscopy was done, and a mass between the vena cava, the azygos vein, and the spine was identified. The procedure was converted to open thoracotomy over the 4^th^ intercostal space. The mass was dissected from the azygous vein, the vena cava, the spine, and the esophagus. Excision was done with hemostasis secured. A chest tube 28 French was inserted. Wound was closed by layers.

Following surgery, the patient was transferred to the ICU. PTH levels dropped significantly reaching to 27 pg/ml. Calcium serum level was back to normal at 8.9 mg/dl. He was transferred the following day to the floor. His postoperative course was uneventful, and he was discharged on the 4^th^ postoperative day. The patient had been followed up in the surgical outpatient clinic for 20 months with no evidence of elevation of PTH or hypercalcemia.

### 2.1. Pathological Findings

**Figure 2:**
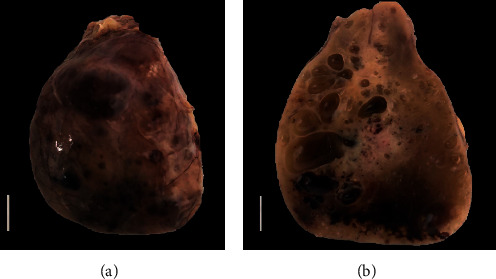
(a) Gross pathological appearance of giant parathyroid adenoma, which weighed 42 grams, featuring intact capsule, cystic areas, and foci of congestion. Bar represents 1 cm. (b) Cut section of the giant parathyroid adenoma featuring tan surface, areas of congestion or hemorrhage, and multiple cystic spaces that were filled with clear fluid. Bar represents 1 cm.

The upper posterior mediastinal tumor consisted of an oval nodular piece of rubbery soft red and tan tissue with attached scant fatty tissue. It weighed 42 grams and measured 6.2 × 4.7 × 2.3 centimeters (Figure 2(a)). On cut section, it showed a rubbery light tan brown surface with areas of hemorrhage, and formation of multiple small cysts ranging from 2-11 millimeters in size (Figure 2(b)).

**Figure 3:**
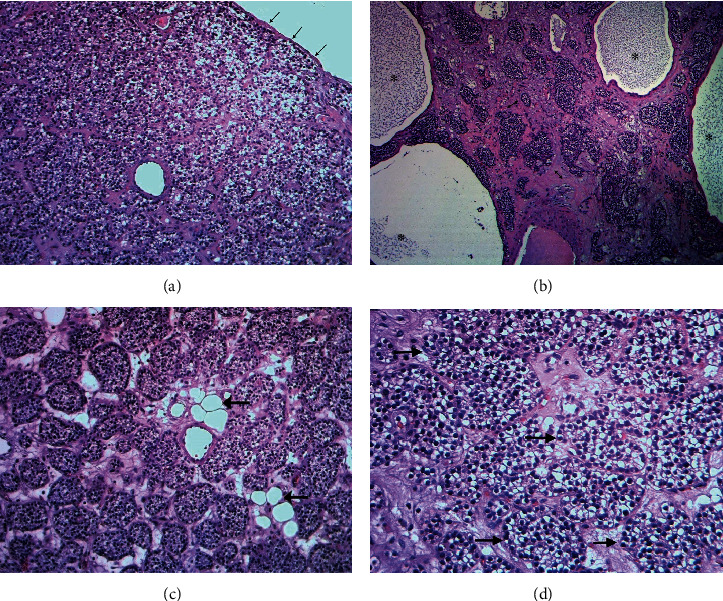
(a) Photomicrograph featuring thin fibrous capsule (arrows) and clusters and nests of chief epithelial cells with clear cytoplasm and rounded monotonous nuclei. The stroma shows vascularized connective tissue (H&E X 40). (b) Photomicrograph featuring tumor nests within fibrotic stroma (double headed arrows) with adjacent cystic areas (asterisk) surrounded by clusters of tumor cells. The cysts are filled with granular secretions. No necrotic tissue is present (H&E X 40). (c) Histological section of parathyroid adenoma featuring clusters and nests of benign epithelial cells with uniform rounded nuclei and water clear cytoplasm. The stroma contains few fat cells (arrows) (H&E X 200). (d) Histological section of parathyroid adenoma featuring clusters and nests of benign epithelial chief cells (arrows) with uniform rounded nuclei and water clear cytoplasm (H&E X 400).

Histological examination revealed the tumor to be surrounded by thin fibrous capsule. It consisted of numerous clusters and sheets of benign parathyroid chief epithelial cells with water clear cytoplasm and monotonous rounded nuclei, free of atypia or significant mitotic activity. Many clusters of tumor cells were embedded within edematous stroma with foci of fibrosis noted. Scattered follicle-like structures or glands filled with eosinophilic material (not colloid) were noted. Very rare clusters of fat cells are noted. Many cystic spaces of variable sizes are noted, some contained eosinophilic material and others were empty; all were surrounded by sheets or clusters of clear cells (Figures 3(a)–(d)). These findings are typical of parathyroid adenoma.

## 3. Discussion

Primary hyperparathyroidism (HPT) is not a rare disease, and it frequently exists as a mild condition lacking the florid renal and skeletal manifestations observed in the earlier literature. This is related to the availability of measurement of serum calcium levels, resulting in an increase in the discovery of cases of hyperparathyroidism (HPT) [2]. Data from the Rochester Epidemiological Project showed that the incidence of primary hyperparathyroidism increased sharply in the United States in July 1974, when the serum calcium level was included in the standard chemistry panel; from 1993 to 2001, the estimated incidence was approximately 22 cases per 100,000 persons per year [3]. Our patient presented with reduced level of consciousness and weakness and was found to have hypercalcemia which leads to further investigation for HPT and the discovery of a PA. Clinicopathological correlates of primary HPT include PA (80–85%), hyperplasia (10–15%), and carcinoma (<1–5%) [4–6].

The size and weight of a parathyroid gland are frequently the only intraoperative determinants of abnormality. The normal weight of the parathyroid gland was found in one study of 100 previously healthy subjects who died suddenly and had a postmortem examination to be 42.6 ± 20.3 mg, with a range of 22-103 mg. Gland weight varied with age, increasing to a maximum in the 41-60-year-old age group [7]. In a study of 240 patients who underwent parathyroidectomy, the mean weight of the adenomas was 553.7 ± 520.5 mg (range: 66–2536 mg) [8].

Giant PA is defined as those with a weight more than 3.5 grams, based on a study of 300 patients with PA who underwent parathyroidectomy for primary hyperparathyroidism. The median weight of PAs in that study was 0.61 grams, with a range between 0.05 and 29.93 grams. The giant adenomas were defined as weight ≥ 95^th^ percentile or 3.5 grams [9].

The parathyroid glands develop from the third and fourth pharyngeal pouches in humans between the fifth and twelfth weeks of gestation. The inferior parathyroid glands originate from the third pouch along with the thymus and are named parathyroids III (PIII). The association of thymus and PIII had been labeled as the parathymus complex. In about 5% of cases, PIII is located in the chest within the retrosternal thymus, its posterior capsule, or in contact with major mediastinal blood vessels. A lower position can result in PIII to be in contact with the pericardium or the pleura [10].

The distribution of the location of PAs can be widespread, located as high as the submaxillary triangle down to the pericardium in one large series of 400 patients from Massachusetts General Hospital, with 84 parathyroid tumors were located in the mediastinum, 67 (16.75%) in the anterior, and 17 (4.25%) in the posterior mediastinum [11].

50 patients with primary hyperparathyroidism were managed at a hospital in Germany, and 56 parathyroid tumors were excised. 33 of these were eutopic, while 23 were ectopic: 10 intrathyroidal, 7 intramediastinal, and 6 retrosternal. The authors defined the intramediastinal tumors as those requiring sternotomy or thoracotomy for extirpation. They reviewed seven series dating from 1941 up to 1974 with a total of 2089 parathyroid tumors. There were 144 tumors located in the mediastinum, ranging from 1.4% to 21% (average 6.9%) of all parathyroid tumors in these series [12].

In a review of 145 cases of primary HPT from Mexico, an ectopic parathyroid location was detected in 13 cases (9%). The authors stated that their patients with primary HPT related to ectopic adenomas had significantly higher serum calcium levels, as in the current case, and larger tumors than patients with eutopic parathyroid glands, and more frequent HPT-related bone disease [13].

The location of ectopic PAs, whether they are giant or nongiant adenomas, in the posterior mediastinum is quite uncommon. In one series, 17 ectopic mediastinal parathyroid adenomas (MPA), none of which were giant adenomas, were located in the anterior mediastinum, and none was observed in the posterior mediastinum [14]. A recent review of ectopic parathyroid adenomas reported between 2009 and 2019 showed that out of 21 cases, 6 were found to be located in the mediastinum. Out of these six cases, only one was located in the posterior mediastinum via CT imaging [15].

A literature review (1980-2012) of MPA showed that the prevalence of ectopic parathyroids from 28% to 42% in autopsy series and from 6.3% to 16% in surgical series of patients operated on for HPT and even up to 45% in patients who went reoperation. The actual prevalence of MPA has been reported to be 6%-30%. They were reported to be associated with more severe clinical manifestations with higher calcium levels, which could be related to delayed diagnosis and localization. Preoperative localization studies are quit important before mediastinal exploration is initiated. Technetium ^99m^Tc-sestamibi scintigraphy had a reported sensitivity of 80-90%. This radiological study was helpful in the current case, and the location of the MPA was determined preoperatively. Ultrasonography has not been proven to be helpful in MPA in contrast to cervical PAs where it is of value. CT and magnetic resonance imaging (MRI) may provide valuable data with regard to its anatomic location. MPAs were rarely diagnosed before surgical exploration, and most patients had mediastinal exploration only after failed neck exploration [16].

The evolving technology in imaging had resulted in improvement in localizing the source prior to surgery. One study showed that advances in radiology are helping in localization of MPA. A comparison is made between ^99m^Tc-sestamibi scintigraphy combined with single photon emission CT (SPECT)/CT and ^18^F-fluorocholine PET/CT (FCH-PET/CT), revealing that FCH-PET/CT offers an equal or even superior detection of MPA [17].

^99m^Tc-sestamibi parathyroid scan (SPS) with SPECT/CT were utilized in 53 patients with primary HPT, and revealed that SPS interpretation method correlated perfectly with the surgical finding in 48/53 patients (90.6%); it was on the correct side of the surgical finding but not the exact location in 3/53 (5.7%) and nonlocalizing in 2/53 (3.7%) [17]. SPS was utilized in localization of the MPA in our case with success, thus helping in the selection of the right surgical procedure (thoracotomy) to excise the tumor. MPAs were rarely diagnosed before surgical exploration, and most patients had mediastinal exploration only after failed neck exploration [18].

Since the earliest scholarly description of pathology of primary HPT by Castleman and Mallory in 1935, adenomas were considered to form the commonest underlying lesion. The two basic criteria for the diagnosis of adenoma were that the lesion be solitary, involving one gland and occasionally two, and that the lesion be surrounded by a rim of parathyroid tissue. Involvement of a single gland was considered to be the most important criterion, inasmuch as a rim of normal parathyroid tissue was demonstrable in only about 50% of the cases [19].

Sporadic HPT is caused by a PA in the majority of cases. Histologically, PAs are well circumscribed, with a thin capsule, and is composed of nests and cords of cells with rich capillary network. The dominant cells are the chief cells. In a minority of cases, oxyphilic cells can dominate. Cystic degeneration can occur in PA, as in the current case. PAs are usually devoid of fat cells, but they show rarely the presence of a few collections of adipocytes as in our case [20].

In a recent review of parathyroid pathology, it was estimated that 80% to 85% of cases of PHP is caused by parathyroid adenoma, followed by primary parathyroid hyperplasia (15%), and parathyroid carcinoma (5%). The authors stressed previously set histopathological criteria for the diagnosis of PAs, namely, being well circumscribed, encapsulated by thin fibrous capsule, and composed of chief cells with clear cytoplasm and round nucleus, arranged within delicate capillary network. If not totally absent, stromal fat is sparse as in the current case. A rim of atrophic normal parathyroid tissue is seen outside the capsule in about half of the cases. The absence of such rim of normal parathyroid tissue does not exclude the diagnosis of PA especially in large lesions, as in the case reported here, because large PA, including giant PA, may have outgrown the preexisting parathyroid tissue [21]. Parathyroid carcinoma is a hypercellular neoplasm with thick fibrous capsule and fibrous trabeculae. Mitoses may be frequent and atypical forms are encountered. Tumor cells can have macronucleoli and foci of necrosis [21]. None of these features are present in the current case.

In a study of 574 parathyroid glands resected at Memorial Sloan-Kettering Cancer Center (MSKCC) in New York, there were 195 adenomas. The authors stated that the current definition of PA is no longer purely histologic (complete circumscription and a rim of parathyroid tissue at the periphery with lack of fibroadipose tissue), but rather includes the effect of gland removal, with intraoperative PTH decrease and subsequent return to normocalcemia and long-term cure [22].

We searched the literature for reports of giant PAs in the posterior mediastinum and found twelve cases, including one case with two giant adenomas [23–34] as well as the current case (Table 1). There were 5 males and 8 females with age range of 39 to 74 years (mean 61.1 years). The weight of the tumor was not stated in four cases, but the size was large and they were labeled as giant adenoma. The weight of the remaining seven cases ranged from 4.24 grams to 190 grams (mean 71.4 grams). All patients had hypercalcemia ranging from 11.22 mg/dl to 19.9 mg/dl (normal range: 8.90-10.70 mg/dl). The parathyroid hormone (PTH) levels range from 234.14 pg/ml to 2234 pg/ml (normal range: 15-65 pg/ml). Three tumors were related to the trachea, six to the esophagus, and one was related to both. No relationship to both organs was specified in the remaining cases. Six patients underwent thoracotomy, two had video-assisted minithoracotomy or mediastinoscopy, and two had transcervical approach or cervical collar incision. No cases of parathyroid carcinoma had been reported in the posterior mediastinum.

Many of the patients who had giant parathyroid adenomas underwent previous surgeries or explorations for excision but were unsuccessful. One study from Mayo Clinic reported 38 patients with parathyroid mediastinal tumors out of 2770 patients who underwent operations for HPT, including four giant parathyroid adenomas. These patients with MPA underwent more than one operation for removal of the tumor. It revealed one previous operation in 26 patients, two in 10 patients, and three in one patient. Two patients had also unsuccessful mediastinal explorations in addition to previous cervical operations [35]. In one study of seven MPA was resected using video-assisted thoracic surgery. All the patients had failed previous cervical explorations [36]. Another report documented a patient with hyperparathyroidism who underwent exploration of the neck and excision of three and half parathyroid glands, all were normal, and PTH level was not lowered. The patient came back eight months later with generalized aches, big brown tumor of the mandible, and a broken leg. MRI revealed a 3 cm mass behind the right subclavian artery and extending to the spine. The mass was excised through sternotomy [37].

A study of 88 patients was operated on for persistent or recurrent disease underwent 98 reoperations. Missed hyperfunctioning parathyroid gland was found on reoperation in normal (eutopic) position in 49 (55.5%) patients and in ectopic position in 39 (44.3%) patients, including 20 (22.7%) cervical and 19 (21.6%) mediastinal. The authors concluded that multidisciplinary treatment team cooperation at a referral center consisting of accurate preoperative localization, expertise in parathyroid reexplorations, and correct use of intraoperative adjuncts contribute to a high success rate of parathyroid reoperations [38]. Another study analyzed the causes and the outcome of reoperations in 38 patients in a series of 1448 patients from France who underwent operation for primary HPT. They found that the causes of reoperations include the following: inexperience of the surgeon who did not perform ectopic location exploration (42%), ectopic locations (75%), unrecognized multiglandular disease (39%), parathyreomatosis (3%), and true recurrence, commonly in MEN or familial non-MEN HPT settings (18%) [39].

A third study of over 700 parathyroidectomies revealed 46 reoperative cases for persistent HPT, with most patients had undergone bilateral neck surgery and yet the abnormal parathyroid glands found at reoperation were present in the neck (91%), with only 7 of these were ectopic either within the thymus gland or the mediastinum. The authors compared the positive predictive value (PPV) of various types of radiologic imaging in these cases and found that ^99m^Tc-sestamibi imaging had 53% for all abnormal glands and 67% for ectopic glands, compared with ultrasonography (57% and 50%) and computed tomography (55% and 50%). They conclude that patients with persistent HPT requiring reoperative parathyroidectomy need additional imaging to localize the culprit parathyroid gland [40].

In conclusion, we present herein a case of a giant PA that presented with deteriorating consciousness due to hypercalcemia. Laboratory workup and ^99m^Tc-sestamibi scintigraphy confirmed the presence of a posterior mediastinal paratracheal ectopic giant PA that weighed 42 grams, which was excised through open thoracotomy. Posterior MPA are quite uncommon with only 14 cases previously reported in the literature. We reviewed these cases and discussed reoperations in HPT which is encountered frequently in ectopic PA as well as the role of radiological studies in localization of these tumors.

## Figures and Tables

**Table 1:** Reported cases of ectopic giant posterior mediastinal parathyroid adenoma.

Author year (reference)	Age/sex	Presentation	Imaging	Tc-99 MIBI	Serum calcium level	Serum PTH level	Surgery	Size	Weight	Follow-up
Hargreaves and Wright [23]	66/F	Change in tone of voice and nonspecific left should pain	Large right retrotracheal posterior mediastinal mass, extending from high in the peritracheal region to below the hilum (chest roentgenogram)	No data	13.23 mg/dl	No data	Right posterolateral thoracotomy	14 × 7.5 × 3.5 cm	190 g	No data
Ogawa et al. [24]	72/F	Hypercalcemia for evaluation	Computed tomographic scan with 3 dimensional reconstruction images revealed a tumor behind the thoracic esophagus	An abnormal accumulation of MIBI at the upper mediastinum	12.5 mg/dl	650 pg/dl	A right-sided thoracotomy was performed to explore the mediastinum. An adenoma situated behind the thoracic esophagus was resected	3 cm	4.25 g	Patient had normal calcium and PTH levels and was discharged on postoperative day 7
Yun et al. [25]	64/F	Abnormal test results	Elongated mass in the posterior mediastinum, extending from the level of the right subclavian artery to the halfway point of the right esophageal wall (CT)	Abnormal uptake in the mediastinum area	13.5 mg/dl	705 pg/dl	Right 6^th^ posterolateral intercostal thoracotomy	12 × 3.5 × 1.7 cm	No data	No recurrence in 2 years
Kiverniti et al. [26]	39/F	Acute stridor, mild dysphagia, arthralgia, mood swings, malaise, and lethargy	Presence of a soft-tissue smooth, solid tumor in the cervical esophagus at the level of the thoracic inlet; the tumor was predominately in the right posterior lateral side (CT)	No data	9.7 mg/dl	No data	Thoraco-cervical approach and a partial longitudinal sternal split	7 × 6 × 5 cm	No data	No recurrence at 3 months follow-up
Çakmak et al. [27]	63/F	Headache and fatigue	Posterior mediastinal mass near the esophagus and trachea (CT)	Revealed mass in mediastinum	13.2 mg/dl	642 pg/ml	Right posterolateral thoracotomy	7 × 5 × 4 cm	145 g	No data
Migliore et al. [28]	65/F	Persistent hypercalcemic syndrome	Presence of a “missed” 7 cm mass in the posterior mediastinum (CT)	Confirmed CT findings	No data	No data	Remedial right video-assisted minithoracotomy	7 cm	95 g	No data
Bayraktar et al. [29]	62/M	Fatigue, unable to walk, and low back pain (lumbalgia)	Bilateral posterior mediastinal masses with a concomitant multinodular goiter (neck ultrasonography)	No significant findings	19.24 mg/dl	3,436 pg/dl (343.6 pg/ml)	Collar incision	Right inferior: 5.5 × 4.5 × 3.5 cm Left inferior: 6 × 4.5 × 3 cm	Right inferior: 74 g Left inferior: 102 g	No recurrence in 12 months
Świrta et al. [30]	52/M	Persistent hypercalcemia	Ultrasound showed single focal lesions below the lower poles of thyroid lobes	An increased accumulation of marker in the right view and the left lower parathyroid gland	2.85 mmol/l (11.4 mg/dl)	1050 pg/ml	Bilateral neck reexploration. Upper posterior mediastinal enlarged parathyroid close to the left recurrent laryngeal nerve was excised in 2010	6 cm in diameter	22.8 g	In 2013, another adenoma was excised. No recurrence in 12 months
Ebrahimpur et al. [31]	67/F	Nausea, vomiting, polydipsia, and polyuria	Solid-cystic lesion in the superior posterior mediastinum, posterior to trachea (MRI)	Persistent radiotracer uptake in the right middle of the mediastinum	13 mg/dl	291 pg/ml	Open thoracotomy	3.3 × 2.4 cm	No data	No recurrence
Zeng et al. [32]	64/F	Chronic generalized ostealgia and hypercalcemia	Abnormal upper-left mediastinal shadow (CT)	Radiotracer accumulation in posterior superior mediastinum near the esophagus	12.79 mg/dl	24.83 mmol/l (234.14 pg/ml)	Video-assisted mediastinoscopy	No data	No data	No data
Miller et al. [33]	53/M	Nonspecific malaise and fatigue	Well defined, bilobed, uniformly enhancing mass to the left of the esophagus, extending from the suprasternal notch and descending in the posterior mediastinum to the level of the carina (CT)	Unusual linear region of increased intensity to the left of the mediastinum	11.22 mg/dl	179.17 pg/ml	Trans-cervical approach	8 × 3 × 3 cm	30.9 g	No recurrence
Nastos et al. [34]	54/M	Nephrolithiasis, hypertension, and severe osteoporosis	Ultrasound did not recognize any pathological findings	Fusion MIBI with SPECT revealed a 6 cm hyperfunctioning retroesophageal parathyroid gland	12.1 mg/dl	277 pg/ml	Conventional 4 cm collar incision	6.5 cm maximal dimension	8.3 g	Postoperatively, calcium and PTH levels were normalized and patient discharged on day 3 post-op
Amr et al. (current case)	74/M	Generalized weakness and reduced level of consciousness	CT scan showed 6 × 4 × 3 cm well-defined lobulated mediastinal paratracheal mass	Right lower parathyroid lesion extending into the mediastinum	19.9 mg/dl	2234 pg/ml	Single port thoracoscopy converted to open thoracotomy over 4^th^ intercostal space. Mass found between vena cava, azygos vein, and the spine	6.2 × 4.7 × 2.3 cm	42 g	No recurrence in 18 months

## Data Availability

The case report data used to support the findings of this study are included within the article.
